# P-934. Evaluation of Empiric Antibiotic Prescribing Compliance with a Local Community-Acquired Pneumonia Algorithm Across a Multi-Hospital Health System

**DOI:** 10.1093/ofid/ofaf695.1137

**Published:** 2026-01-11

**Authors:** Joel Kennedy, Christie M Bertram, William Justin Moore, Sarah Sutton, Richard G Wunderink, Chiagozie Ifeoma Pickens, Nathaniel J Rhodes, Erin Weslander

**Affiliations:** Northwestern Memorial Hospital, Chicago, Illinois; Northwestern Memorial Hospital/Rosalind Franklin University of Medicine and Science, Chicago, Illinois; Northwestern Medicine, Chicago, Illinois; Northwestern University, Chicago, Illinois; Northwestern University Feinberg School of Medicine, Chicago, IL; Northwestern University, Chicago, Illinois; Midwestern University, Downers Grove, IL; Northwestern Memorial Hospital, Chicago, Illinois

## Abstract

**Background:**

The 2019 IDSA/ATS CAP guidelines recommend empiric *Pseudomonas aeruginosa* (PsA) coverage for patients with prior resistant Gram-negative infections or locally validated risk factors.^1^ In 2017, the NM Health System implemented a CAP treatment algorithm adapted from Shindo et al., stratifying patients by the number of risk factors for drug-resistant pathogens: those with 0-2 risk factors are recommended standard therapy, while those with ≥ 3 are recommended empiric anti-PsA coverage (Figure 1).^2^ The impact of this tool on prescribing patterns across the system remains unclear.

**Figure:**
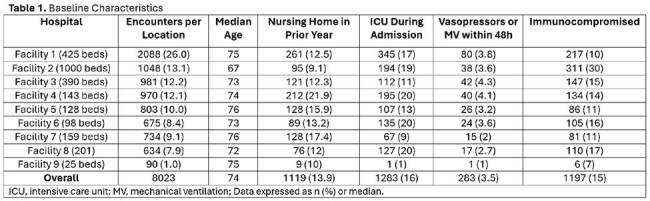

**Figure:**
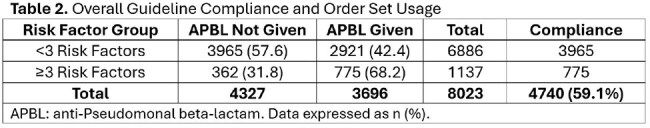

**Methods:**

This IRB-approved (STU00222641), retrospective cohort study included adults admitted to 9 NM hospitals from 10/1/22-9/30/24 with an ICD-10 discharge diagnosis of CAP who received antibiotics within 48 hours of admission and had CAP or CAP with risk factors selected in order entry, which is integrated with the institutional algorithm. Patients with cystic fibrosis, bronchiectasis, or death within 48 hours of admission were excluded. De-identified EHR data were used to assess demographics, comorbidities, microbiology, and antibiotic prescribing (Table 1). The primary outcome was the proportion of cases concordant with the algorithm. Descriptive statistics were used to report counts and percentages.

**Figure:**
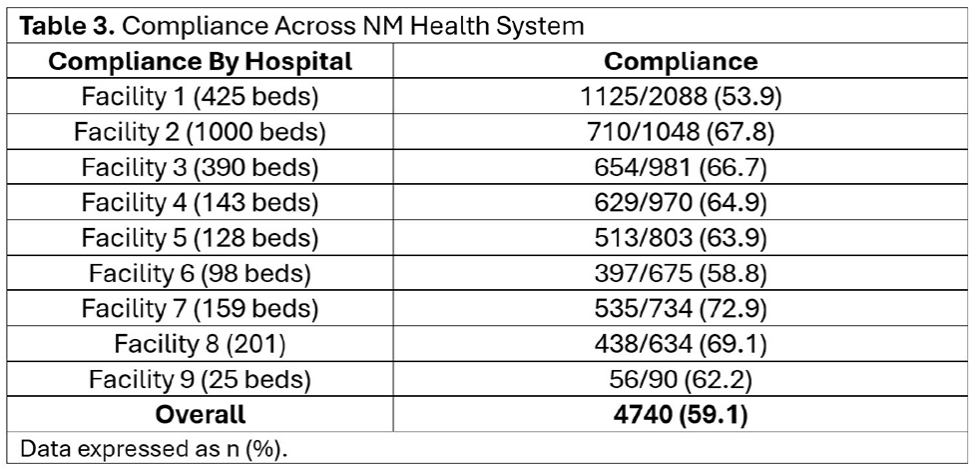

**Figure:**
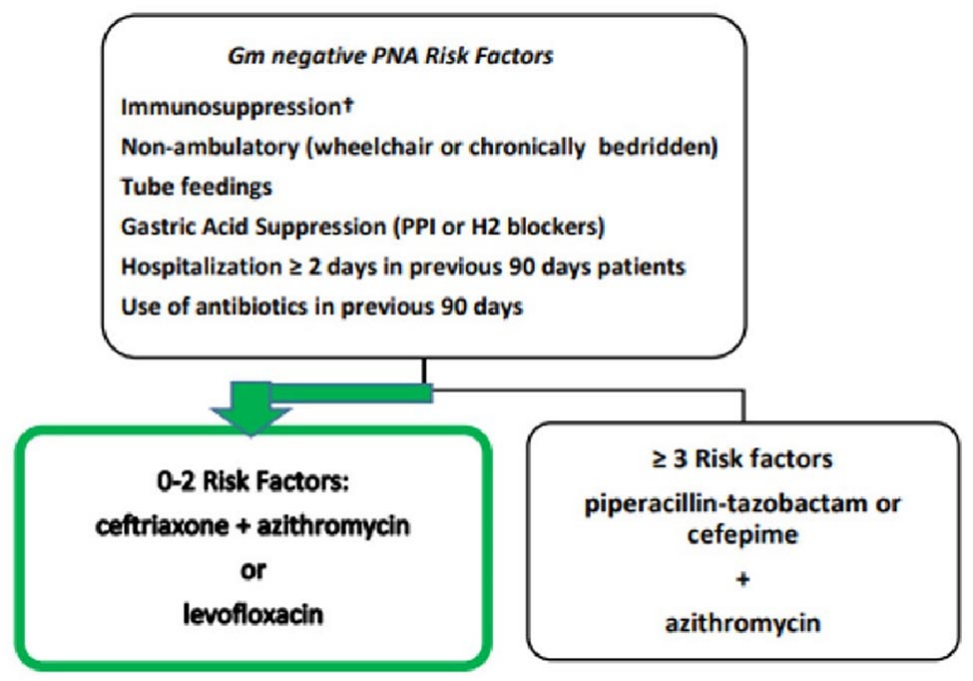

**Results:**

Among 8,023 patients, 59.1% received guideline-concordant empiric therapy (Table 2). Of those prescribed anti-PsA beta-lactams, 42.4% (n=2921/6886) did not meet criteria. Among patients with ≥ 3 risk factors, 68.2% (775/1137) appropriately received anti-PsA-beta-lactam per the algorithm. Compliance varied by site (50% to 69%) (Table 3). CAP order set use was low: 19.9% among compliant cases compared to 15.8% in non-compliant cases.

**Conclusion:**

This study demonstrated suboptimal compliance with a locally developed algorithm guiding empiric therapy for CAP patients at risk for PsA. Variation across sites and limited order set use reflected real-world implementation challenges. Some cases may have been misclassified due to incomplete EHR history in patients without established care within the health system. Further evaluation of the risk stratification criteria and their clinical utility is warranted to inform future stewardship interventions.

**Disclosures:**

Nathaniel J. Rhodes, PharmD MS, Apothecademy, LLC: Advisor/Consultant

